# The polarization of politics and public opinion and their effects on racial inequality in COVID mortality

**DOI:** 10.1371/journal.pone.0274580

**Published:** 2022-09-15

**Authors:** Adeline Lo, Héctor Pifarré i Arolas, Jonathan Renshon, Siyu Liang

**Affiliations:** 1 Department of Political Science, University of Wisconsin-Madison, Madison, Wisconsin, United States of America; 2 La Follette School of Public Affairs, University of Wisconsin-Madison, Madison, Wisconsin, United States of America; 3 Department of Political Science, University of California-Los Angeles, Los Angeles, California, United States of America; Vanderbilt University, UNITED STATES

## Abstract

Evidence from the early months of the COVID-19 pandemic in the U.S. indicated that the virus had vastly different effects across races, with black Americans faring worse on dimensions including illness, hospitalization and death. New data suggests that our understanding of the pandemic’s racial inequities must be revised given the closing of the gap between black and white COVID-related mortality. Initial explanations for inequality in COVID-related outcomes concentrated on static factors—e.g., geography, urbanicity, segregation or age-structures—that are insufficient on their own to explain observed time-varying patterns in inequality. Drawing from a literature suggesting the relevance of political factors in explaining pandemic outcomes, we highlight the importance of political polarization—the partisan divide in pandemic-related policies and beliefs—that varies over time and across geographic units. Specifically, we investigate the role of polarization through two political factors, public opinion and state-level public health policies, using fine-grained data on disparities in public concern over COVID and in state containment/health policies to understand the changing pattern of inequality in mortality. We show that (1) apparent decreases in inequality are driven by increasing total deaths—mostly among white Americans—rather than decreasing mortality among black Americans (2) containment policies are associated with decreasing inequality, likely resulting from lower relative mortality among Blacks (3) as the partisan disparity in Americans who were “unconcerned” about COVID increased, racial inequality in COVID mortality decreased, generating the appearance of greater equality consistent with a “race to the bottom’’ explanation as overall deaths increased and substantively swamping the effects of containment policies.

## Introduction

Scientists and the public have quickly coalesced around an understanding of the pandemic and its effects that emphasizes its disproportionate impact on racial minority groups in the United States. The clear and intuitive story that emerged from early research was that, far from being a great “leveler” affecting all equally, minorities in the U.S. were, for a variety of reasons, more likely to suffer from COVID-19, with higher infection rates, severe illness and mortality [1].

New data suggests that our understanding of the racial inequities in mortality from COVID-19 must be revised. The CDC wrote in April 2021, that “though disparities remained,” racial and ethnic variation in COVID’s impact “became less pronounced as the pandemic spread throughout the country” [2], and more recent work has confirmed the “convergence” between black and white COVID-19 mortality over time [3] while noting that geographic spread of the disease to new regions cannot fully account for the pattern by itself.

A pressing question thus emerges: How can we explain *shifting* patterns in COVID mortality inequality given that initial explanations highlighted the impact of *static* factors such as geography [4], pre-COVID institutional inequalities in healthcare/income and historical segregation in the U.S. [5]?

Addressing this question solely through a focus on biomedical or epidemiological factors is insufficient: even when considering the state of public health systems, vaccine productions and distribution, etc., “the biggest problems…are political” [6]. Thus, any answer to these puzzles must reckon with two central facts: that “governments play a central role in combatting pandemics” and that individual behavior and responses—largely influenced by political beliefs and ideology—are also of critical importance [7]. Researchers have collected mounting evidence on the latter point, from how partisanship predicts social gathering behaviors [8] to partisan differences in prevention behaviors [9] and social distancing [10], throughout the course of the pandemic. Viewing the causes of COVID-19 mortality inequality through the lens of politics and beliefs focuses our attention on political factors at the level of government action—mandates, containment and suppression policies—and mass public opinion—particularly the “partisan spread” in concern over coronavirus.

Answering these questions require data fine-grained enough to capture temporal and geographic variation in race-based mortality due to COVID-19, total mortality patterns, changing policy adoptions, and fluctuations in public opinion on both sides of the partisan aisle. Accounting for the temporal aspect is particularly important as the puzzle is about shifts in patterns of racial inequality in mortality *over time*; near-static contextual factors, such as health care systems, age-structures or local urbanicity within races, are important—and likely complementary to our posited political forces—but cannot explain all the variation in time-varying inequality. To account and control for such time-invariant factors, we rely on a fixed effects estimation (intercepts for states, time) applied to a panel dataset. Our data is observed at the state-week level and is comprised of measures of racial inequality in COVID mortality and total COVID mortality (CDC), an index of government policies enacted for containment and health purposes (Oxford Covid-19 Government Response Tracker (OxCGRT) [11]), and public opinion data, across parties and racial groups, over outbreak concerns of COVID in respondent-local areas [12] (Civiqs) (see materials & methods).

## Results

**Fig 1:**
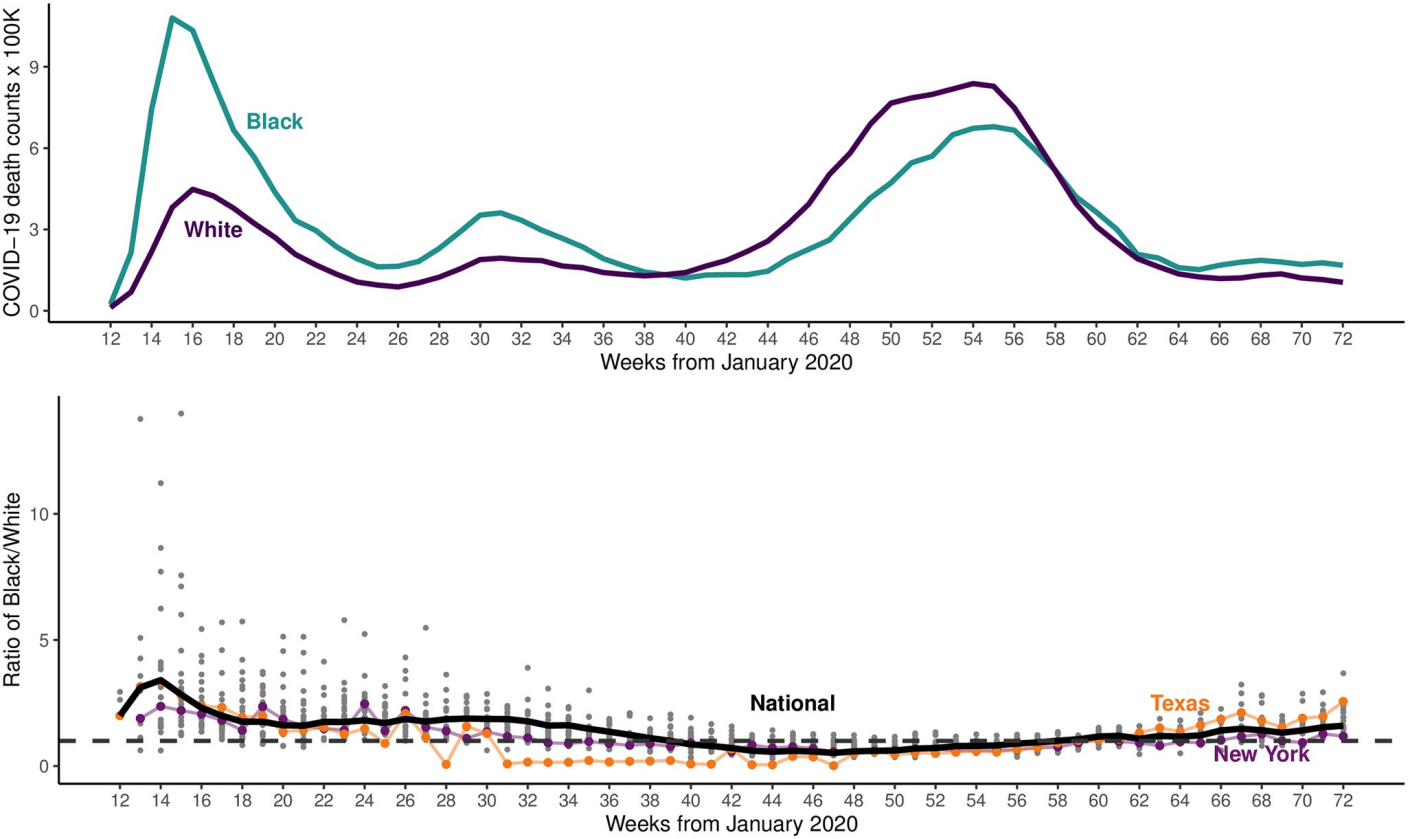
COVID mortality per capita by race (top); black-divided-by-white (bottom) COVID deaths per capita times 100,000.

**Fig 2:**
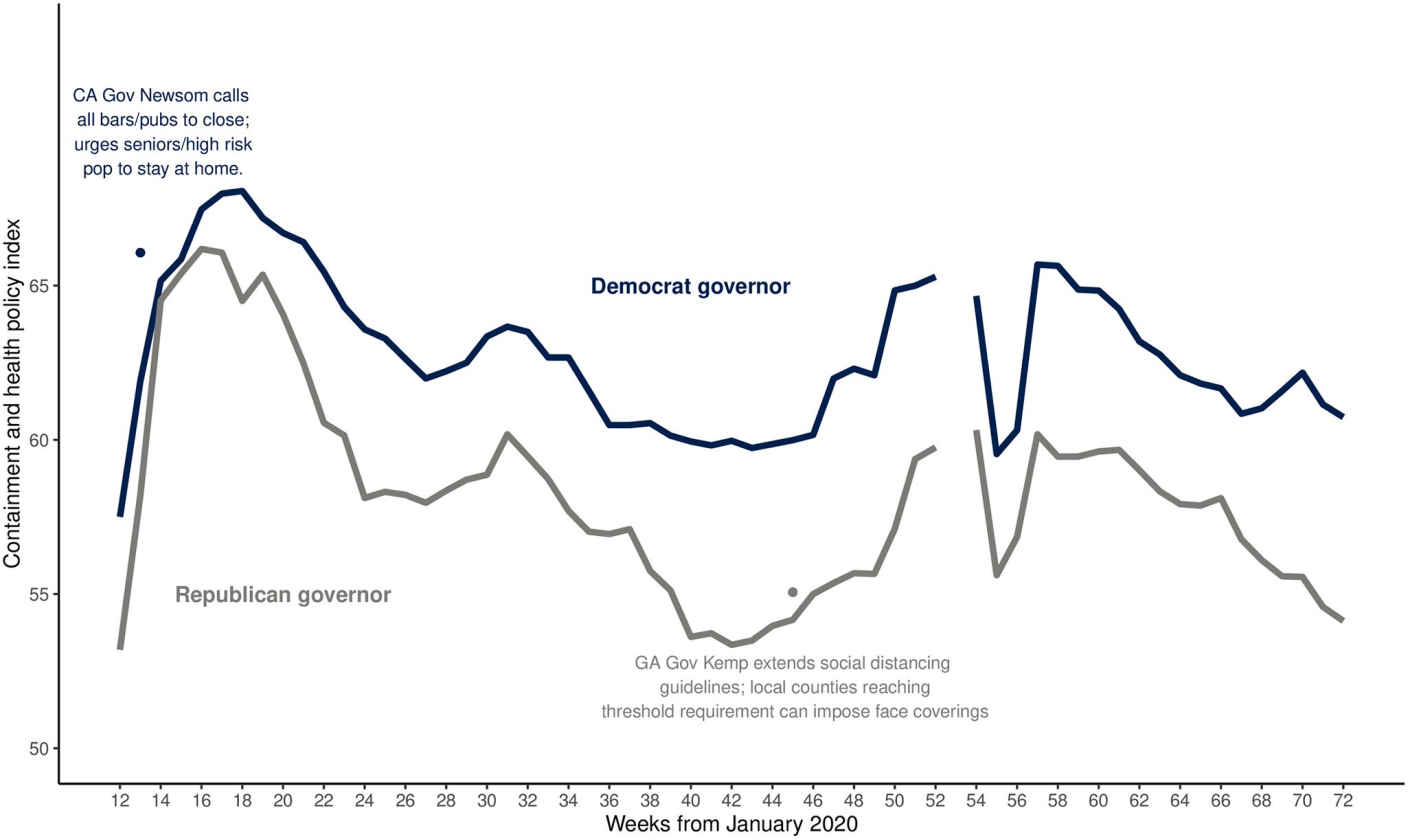
Containment and health policy adoption by governor party.

**Fig 3:**
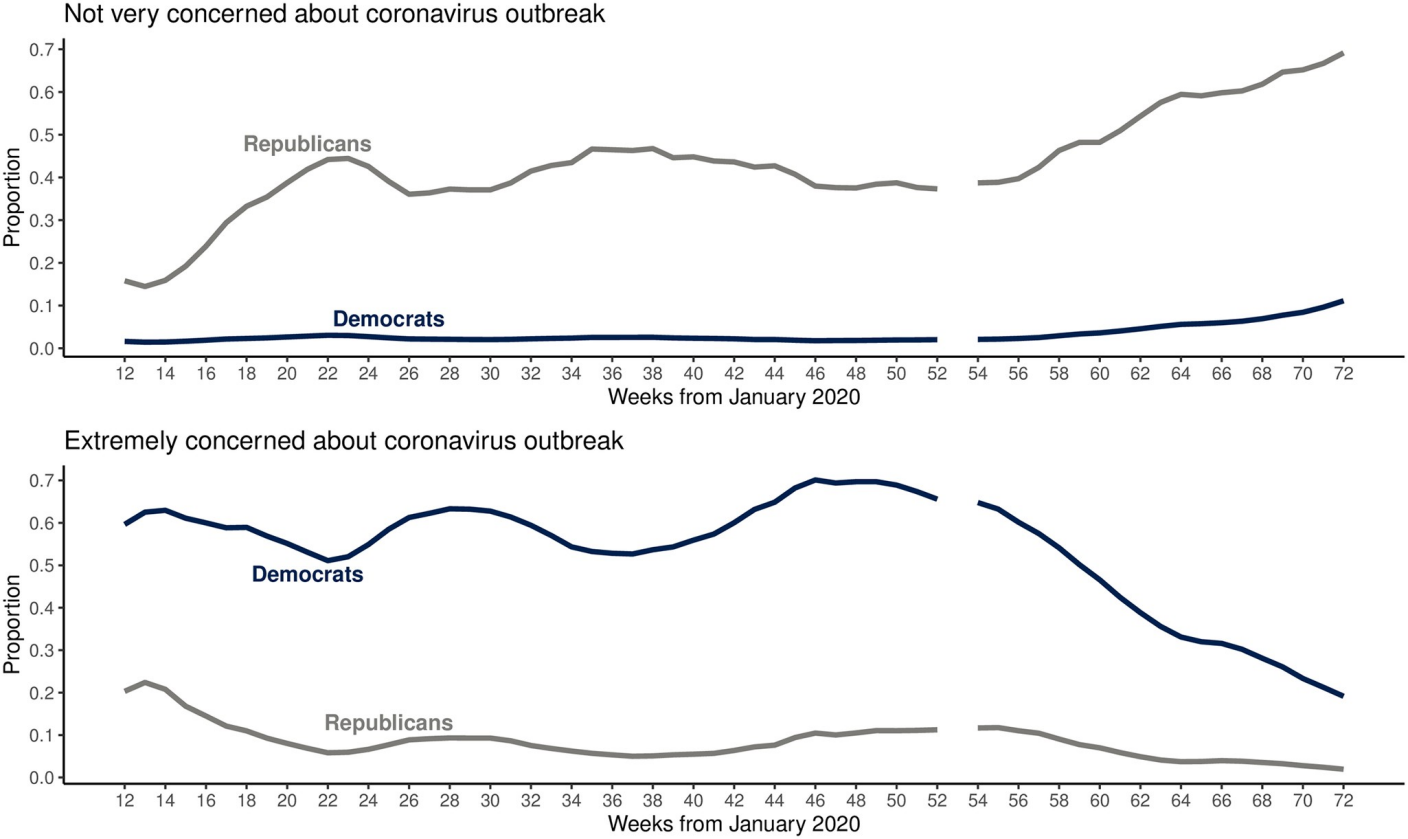
Public concern about local outbreak of COVID by party affiliation, for not concerned and extremely concerned.

Fig 1 (top) presents the components to our ratio outcome measure—COVID-19 mortality per capita × 100,000 death counts (non-age standardized)—from January 2020 to May 2021 for black and white Americans as well as the ratio itself (bottom). Mortality has varied generally and between races over time, beginning with wider differences between black and white mortality in Q1 of 2020 (larger black compared to white mortality in Weeks 12 to roughly 38) that have converged over time, with increasing white mortality over time as major driver of this convergence (white mortality increasing with respect to black in Weeks 39–58). However, despite the appearance of the raw data, our analysis confirms that accounting for their comparatively younger general population (with age-standardization), black Americans still shoulder a comparatively greater mortality burden [13]. Fig 2 depicts a key predictor, containment and health policies enacted at the state level, showing that efforts to curtail the pandemic have varied significantly, with Republican Governor-led states adopting later and fewer policies compared to their Democratic counterparts [14]. Finally, Fig 3 visualizes “concern about COVID” among the American public, demonstrating that as the pandemic continued, a sharp partisan divide emerges and grows over time. Our thesis is that the political divide in concern over COVID is a significant causal factor in driving the observed patterns of racial inequality in COVID mortality.

**Fig 4:**
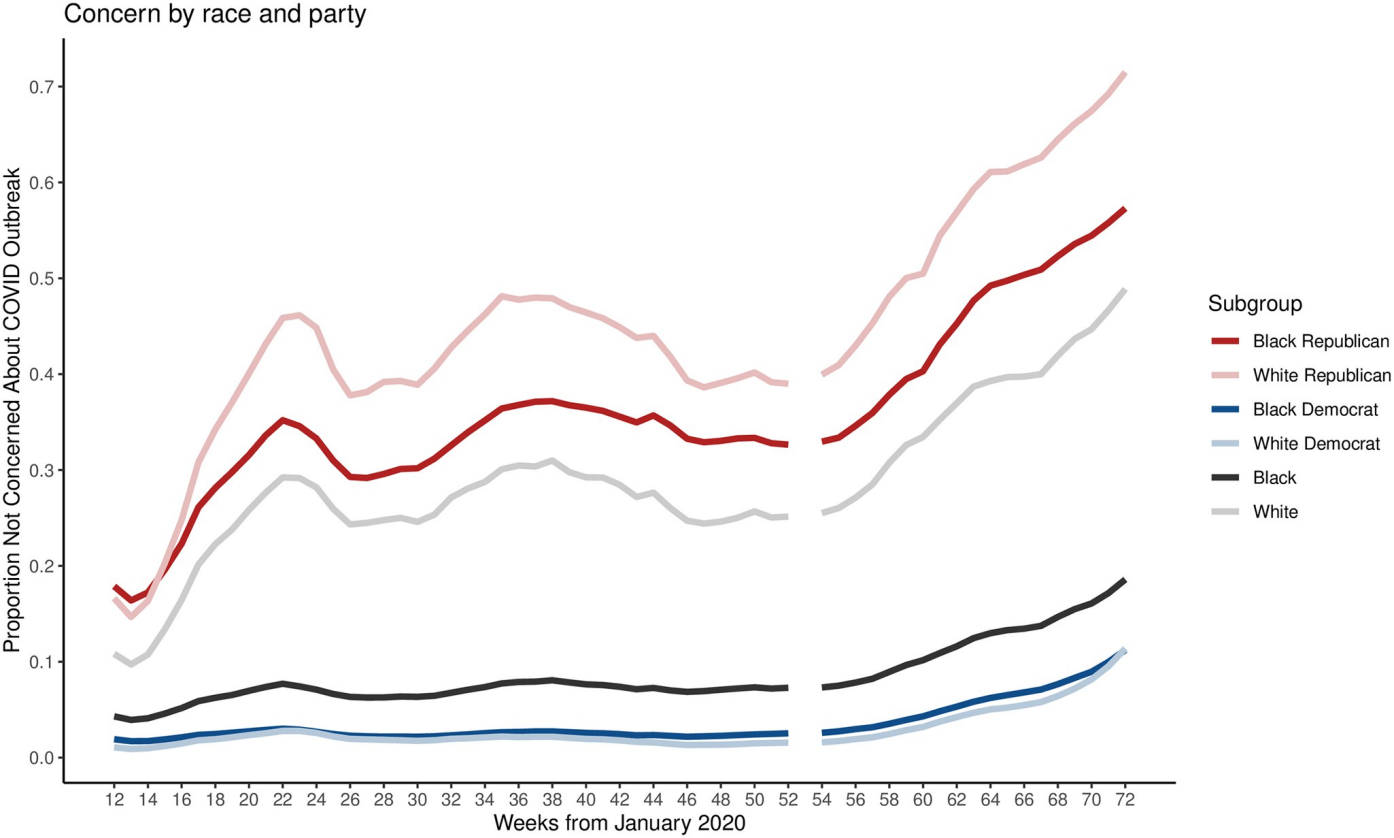
Public concern about local outbreak of COVID-19 by party and race. Differences in proportion of Americans not concerned with COVID-19 by party (holding race constant) are larger than differences in proportions by race (holding party constant), suggestion partisan disparities in concern dominate racial disparities.

An alternative conjecture is that racial disparities of opinion are more influential than partisan differences. If that were the case, then within-party differences (across race) ought to be larger than across-party differences (by race). Fig 4 shows that the opposite is true: within-party differences in opinion (by race) are small and swamped by differences across-party. This is suggestive of overall differences in opinion being driven by party affiliation rather than race and differences in opinion across races being driven by party affiliation, not through any inherent differences in concern across races. In our main models, we focus on partisan disparities in concern as a key predictor, though in secondary models include both party and race disparity measures.

We follow [3] and operationalize racial inequality in COVID-19 mortality as a ratio of black-divided-by-white mortality (ratio of two trend lines in Fig 1). As levels of overall mortality are plausibly causally related to inequalities in racial mortality [3]—a “race to the bottom” effect, whereby the virus spreading everywhere directly increases black *and* white mortality such that the ratio approaches one—we include a measure of total COVID-19 mortality at the state-week level to account for this causal pathway. And although innumerable combinations of suppression and containment policies are available to policymakers, in practice they are bundled and collinear (e.g., stay at home policies are often paired with masking mandates). As such, we use a single index of containment/health policies constructed by OxCGRT (0 to 100, for level of state response), with a rolling average of two weeks to allow for delayed impacts of policies. We use two week lag times given this is identified as the time span between onset of COVID symptoms and occurrence of COVID-19 death as well as between policy implementation and effects on COVID-19 outcomes [15, 16]. In robustness checks we utilize three and four week lags in the main model which result in similar substantive findings. Finally, we measure public opinion about COVID—and specifically *partisan divide* in levels of concern—through a ratio of the proportion of Democrats who have *no concern* about COVID-19 over the proportion of Republicans who have *no concern*. A similar ratio is created for *extreme concern*. In a second model (Model 2), we also include parallel measures for racial disparities in COVID-19 concern and no-concern.

Three notable results emerge from our analysis (Table 1). First, in our main model (Model 1) we find that total mortality is negatively correlated with racial inequality in mortality, consistent with a “race to the bottom” explanation in which a greater number of overall deaths result in (only) a semblance of greater equality. Second, containment and health policies are overall negatively associated (-0.011, p = 0.031) with racial inequality in mortality, likely resulting from lower *relative* mortality among Black Americans. That is, despite the fact that individual policies may benefit or only be possible/followed for certain racial groups (encapsulated in the phrase,“social distancing is a privilege”; see as an example [17], for a discussion on differences in vaccine effectiveness across racial groups), their net effect is to reduce inequality. Finally, as partisan disparity in Americans “unconcerned” about COVID increases (such as the growth of the proportion of Republicans unconcerned seen in Fig 3), racial inequality in COVID mortality decreases as a result of greater numbers of white Americans dying. This effect is significant (p = 0.005), positive (13.094), and (statistically significantly) larger than the effect of relative amounts of high levels of concern among the public. Inequality in COVID mortality is thus much more directly caused by the relative numbers of partisans who evince *no concern at all* about COVID than the number of people who express higher levels of concern.

**Table 1:** Main model estimation results.

	Racial inequality in COVID-19 mortality	
	Model 1	Model 2
Total COVID-19 mortality	-0.022 *(0.012)	-0.015(0.015)
# Black (vs White)		-4.658(7.513)
who report “No Concern”		
# Black (vs White)		0.587 **(0.276)
who report “Extreme Concern”		
# of Democrats (vs Republicans)	13.968 ***(4.464)	20.572 ***(7.607)
who report “No Concern”		
# of Democrats (vs Republicans)	-0.091(0.083)	-0.153 *(0.092)
who report “Extreme Concern”		
Policy index	-0.012 **(0.005)	-0.013 **(0.005)
State & Week FE	Yes	Yes
Observations	1,225	1,225
F Statistic	12.003***	11.517***
	(df = 4; 1131)	(df = 6; 1129)

*Note*:

*p<0.1;

**p<0.05;

***p<0.01

Finally, we find that although there is a small part of the narrative explained purely by race, it is swamped by partisan politics. In Model 2, which includes additional controls for racial concern for COVID-19, our results for partisan differences in concern remain substantively similar. Here, we do find that the proportion of Black (vs White) Americans who report extreme concern for COVID predicts racial inequality in mortality, but that estimate (0.587) is nearly forty times smaller than our estimate of the effect of partisan disparities in levels of concern (20.572).

Our data and estimation strategy also allow us to consider counterfactual thought experiments: what if states enacted policies in more similar, less partisan-divided, ways? What if the partisan disparity in COVID concern decreased? We estimate that if Democratic governors behaved more similarly to their Republican counterparts—by enacting fewer containment policies and doing so in a more delayed fashion—racial inequality in COVID mortality would increase by 13.48% (Table 2), a larger jump than seen in late March 2020 (weeks 13–14 in Fig 1). Additionally, if the number of Democrats (compared to Republicans) who were “unconcerned” about COVID increased by 10%—roughly equivalent of “purple” Minnesota looking more like “red” Alabama in partisan concern—during the peak of the first wave of the pandemic, racial inequality in COVID mortality would increase substantially (+19.07%).

**Table 2:** Counterfactual scenarios.

**Scenario 1**	
Democrat governors more similar to Republican governors in	
% change in containment & health policy index	-3.54
% change in racial inequality in COVID mortality	+13.48
**Scenario 2**	
Number of Democrats who are “unconcerned” about COVID rise	
% change in Democrats unconcerned/Republicans unconcerned	-10
% change in racial inequality in COVID mortality	+19.07

## Discussion

Our contribution lies in addressing the puzzle of shifting patterns of COVID mortality inequality. Apparent reductions in inequality are predominantly driven by increasing numbers of COVID-related deaths among white Americans rather than decreasing mortality among black Americans. Two political factors are key to understanding this pattern: state-instituted public health policies to combat COVID and levels of concern (by party) about COVID-19 among the public. Adoption of more containment policies is associated with less racial inequality in mortality; that Republican governors adopted fewer policies and with significant delays contributed to their states’ experienced racial inequalities in mortality. And, as partisan disparity in Americans who were *not concerned at all* about COVID outbreaks grew during the course of the pandemic, racial inequality in mortality decreased (through increases in white American deaths).

We explored how political *and* racial disparities in opinions affect racial inequalities in COVID mortality, demonstrating that partisan differences remain the dominant force in two ways: first, by illustrating in Fig 4 that party differences in opinions about COVID are more substantial than across-race differences in opinions and second, estimating a second model that includes measures of racial disparities in opinion and finding that partisan differences in opinion remain large in magnitude and statistically significant. Ultimately, disparity in opinions are likely to relate to differential preventative behaviors around COVID-19 in that preventative behaviors are influenced by opinions about how dangerous COVID is—which in turn can be influenced by race and political affiliation. We present both such dimensions for disparities in opinions and contend that, of the two, partisan differences appear to be the driving force in affecting inequality in mortality.

One open question is whether our partisan concern effects are acting through infection rates and conditional-on-infection mortality rates. While evidence on infection rates should be taken with care (given the level of under-reporting, particularly early in the pandemic), it indicates that infection rates were higher for Black than for White Americans. Over time, this changed and White infection rates became higher (during the period of our study) than those of Black Americans [18]. Thus, given the known higher COVID-19 related age-adjusted mortality of Black (over White), it is highly likely that our effects are the result of higher infection rates. However, given that the measurement of cases is highly controversial (even more so in the early months of the pandemic), we remain cautious about this interpretation.

Taken together, our findings suggest caution in over-emphasizing “equality” in racial mortality given that observed increases in equality in black/white mortality have resulted from higher levels of overall death rather than a reduction in risk for racial minorities. Political polarization has affected racial inequality in COVID outcomes through top-down and bottom-up avenues, with evidence that the latter may play a comparatively greater role. As our data ends at 2021 Q2, open questions that remain include how on-going vaccine rollouts—similarly linked to partisan differences in uptake and historically-founded medical mistrust among racial minorities [19, 20]—may further shape emerging patterns of racial inequality in mortality and how results of the 2021–22 U.S. elections may shift policies across states.

## Methods

Data are from OxCGRT [11], Center for Disease Control (CDC) COVID-19 data tracker [21], Civiqs [12], CDC WONDER [22], to track the U.S. panel coverage of policies data, COVID-19 mortality data, public opinion during the pandemic, and population by state and race, respectively.

OxCGRT data provides a containment and health index that includes closings of workplaces, schools and universities, canceling of public events and limitations of gatherings, closures of public transports, shelter-in-place orders or stay-at-home orders, records restrictions on internal and international movements, records presence of public information campaigns and government policies on access to testing and contact tracing, includes face mask policies, and policies for the protection of the elderly. OxCGRT’s index is an average of the above individual component indicators.

COVID-19 mortality deaths are defined by the CDC as “directly from death certificates filed at the state and local level, and feature counts of COVID-19-related deaths by age, gender, race and Hispanic origin, place of death, and include information on other health conditions and comorbidities involved in these deaths” [21]. Total mortality in this report refers to the total number of COVID-19 deaths.

Civiqs owns and operates a large, nationally representative online survey panel, conducting a large number of interviews on a daily basis; respondent opinions over COVID concern were collected across fifty U.S. states throughout the study period by Civiqs, then aggregated by level of concern to the state level and given for analysis to the authors by state-week, and by (respondent self-reported) race and (respondent self-reported) party.

For additional information, see ref. [13]. To handle CDC-suppressed COVID death counts, we restrict our analysis to states reporting black American deaths in more than 20% of the panel; this excludes states with low proportions of black Americans, resulting in 2088 state-week observations. In the remaining 30 states, weeks which still report NAs due to threshold suppression do not constitute a significant portion of the deaths due to COVID (91% of total COVID deaths are covered). We focus on black to white inequalities in this study; black Americans are a key minority group that has been disproportionately affected by COVID and consistently covered in COVID data efforts [1]. The main model estimated is below:

$$\begin{array}{cc} {\text{Inequality}}_{s,t} & =\alpha_{s}+\gamma_{t}+\beta_{1}\text{Overall}\;{\text{Mortality}}_{s,t}\\ & +\beta_{2}{\text{Policies}}_{s,t-2}+\beta_{3}\text{Partisan}\;{\text{Opinion}}_{s,t-2}+\varepsilon_{s,t} \end{array}$$

Standard errors estimated robust to heteroskedasticity, cross-sectional and serial correlation.

## Supporting information

S1 Text (Txt)

S1 Fig COVID mortality (aged standardized) for black and white Americans from January 2020 to February 2021. Month since January 2020 in the x-axis; y axis is age standardized COVID death rate.
(TIF)

S2 Fig Age-adjusted COVID mortality, by Democrat and Republican governor states from 2020 quarter 1 to 2021 quarter 1. x axes represents year quarter, starting from 2020 Q1 to 2021 Q1; y axes is age standardized COVID death rate.
(TIF)

S3 Fig Per capita times 100,000 COVID-19 mortality rates for states. x axes refers to Weeks from January 2020, and y axes refers to Covid-19 death counts.
(TIF)

S4 Fig Ratio between black and white American COVID-19 mortality per capita. x axes are weeks from January 2020, y axes are COVID death ratios.
(TIF)

S5 Fig American who are extremely concerned about the Covid outbreak in the state level. x axes are weeks from January 2020, y axes are proportions of those who are extremely concerned about the outbreak.
(TIF)

S6 Fig American who are not very concerned about the Covid outbreak in the state level. x axes are weeks from January 2020, y axes are proportions of those who are not very concerned about the outbreak.
(TIF)

S7 Fig Containment and health policy adoption in Democratic states. x axes are weeks from January 2020, y axes present policy index.
(TIF)

S8 Fig Containment and health policy adoption in Republican states. x axes are weeks from January 2020, y axes present policy index.
(TIF)

S9 Fig All states represented in the full data. Shaded states include low/suppressed deaths that do not constitute a part of the main analysis as a result.
(TIF)

S1 File (PDF)
