# Supplementary figures and images for: The polarization of politics and public opinion and their effects on racial inequality in COVID mortality
Source: PLoS One. 2022 Sep 15;17(9):e0274580. doi: 10.1371/journal.pone.0274580 (PMC9477310; doi:10.1371/journal.pone.0274580)

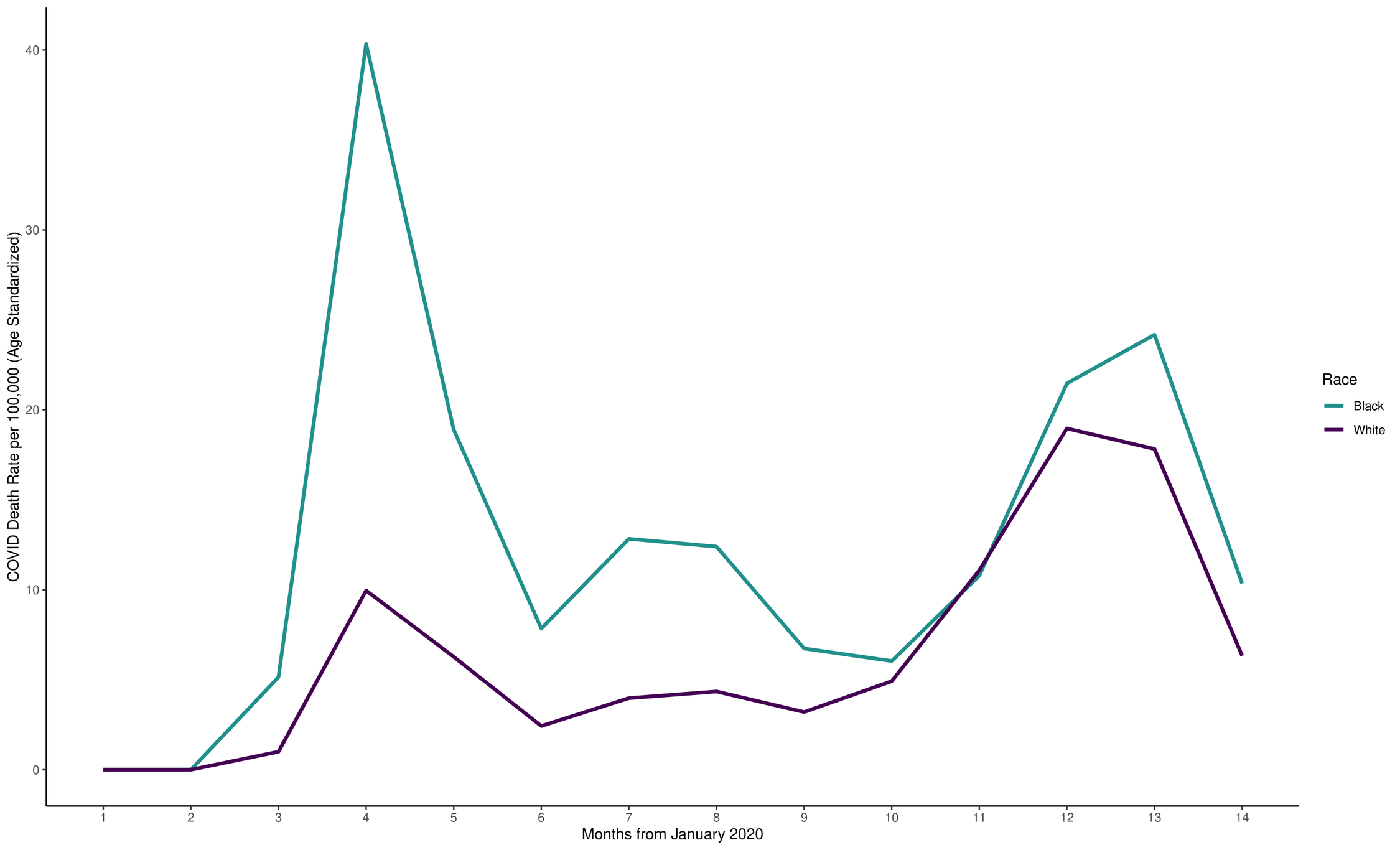

Supplement: S1 Fig — Month since January 2020 in the x-axis; y axis is age standardized COVID death rate. (TIF) [file pone.0274580.s002.tif]

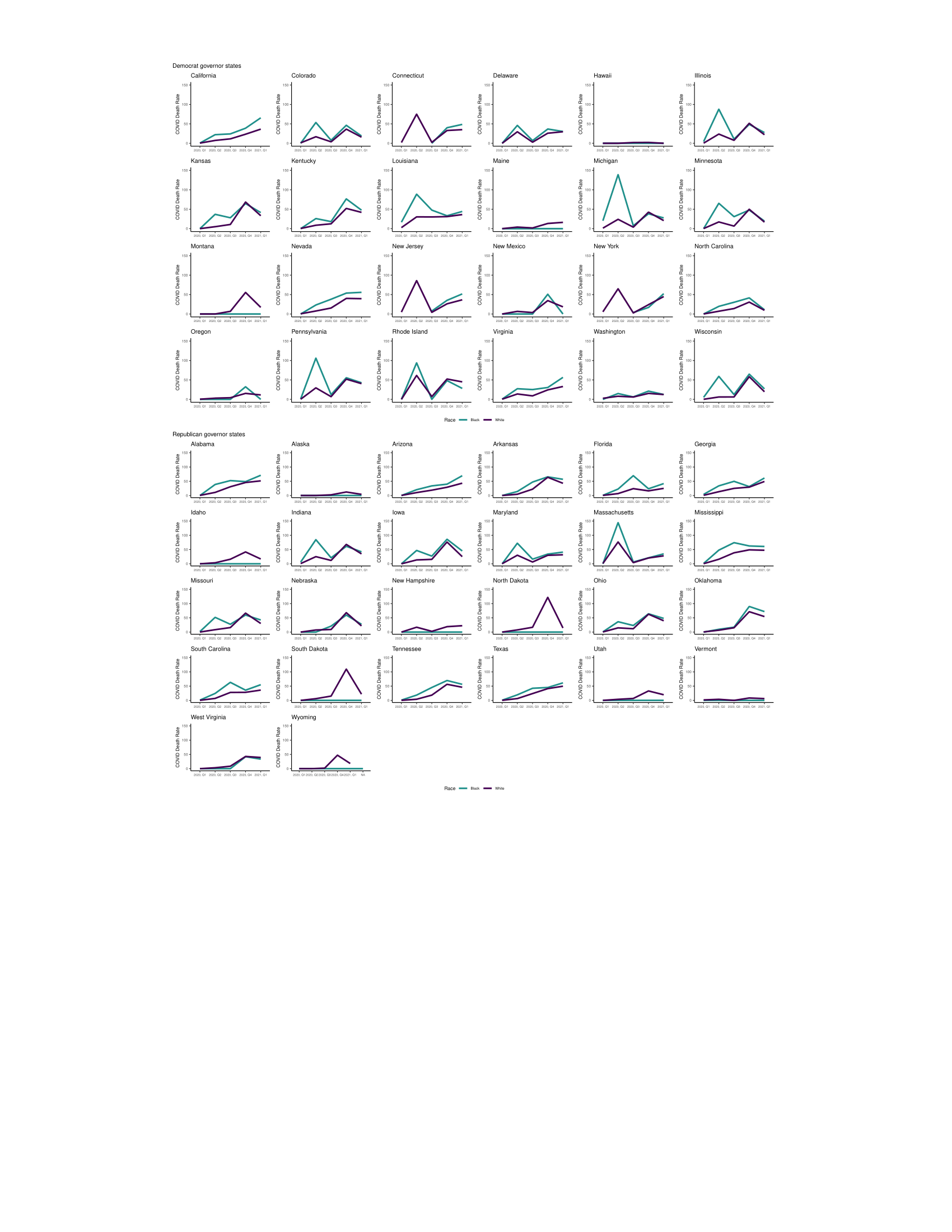

Supplement: S2 Fig — x axes represents year quarter, starting from 2020 Q1 to 2021 Q1; y axes is age standardized COVID death rate. (TIF) [file pone.0274580.s003.tif]

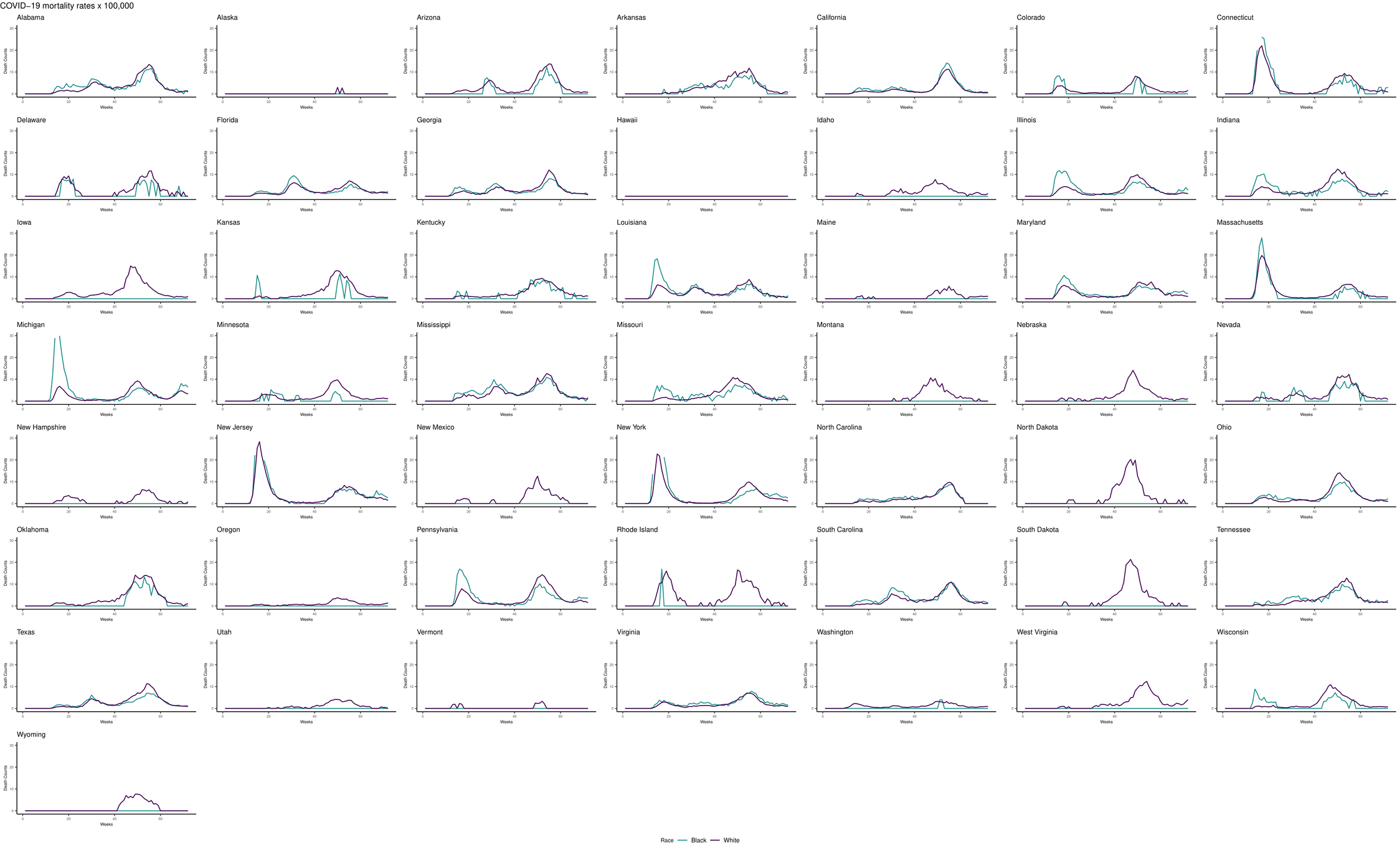

Supplement: S3 Fig — x axes refers to Weeks from January 2020, and y axes refers to Covid-19 death counts. (TIF) [file pone.0274580.s004.tif]

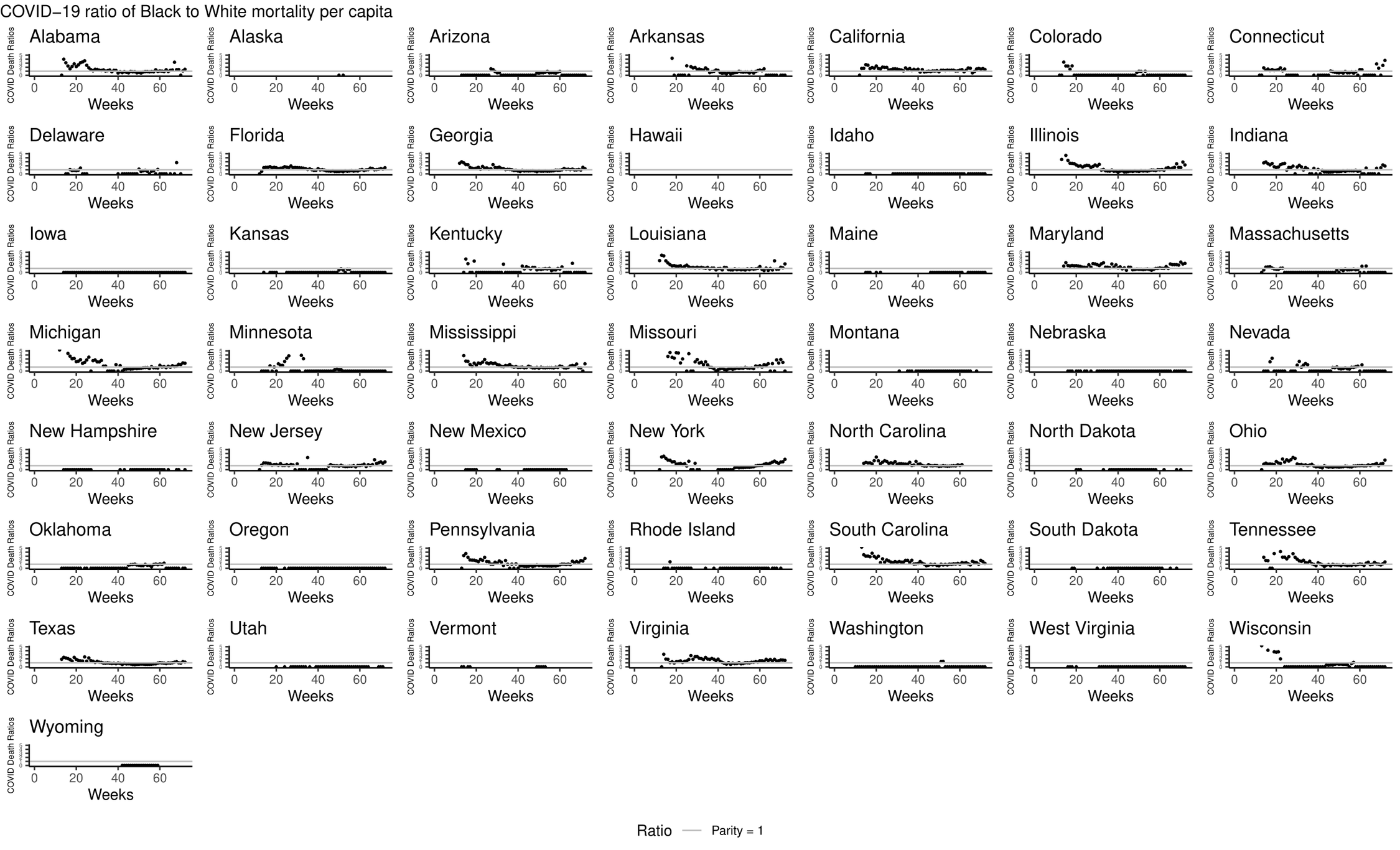

Supplement: S4 Fig — x axes are weeks from January 2020, y axes are COVID death ratios. (TIF) [file pone.0274580.s005.tif]

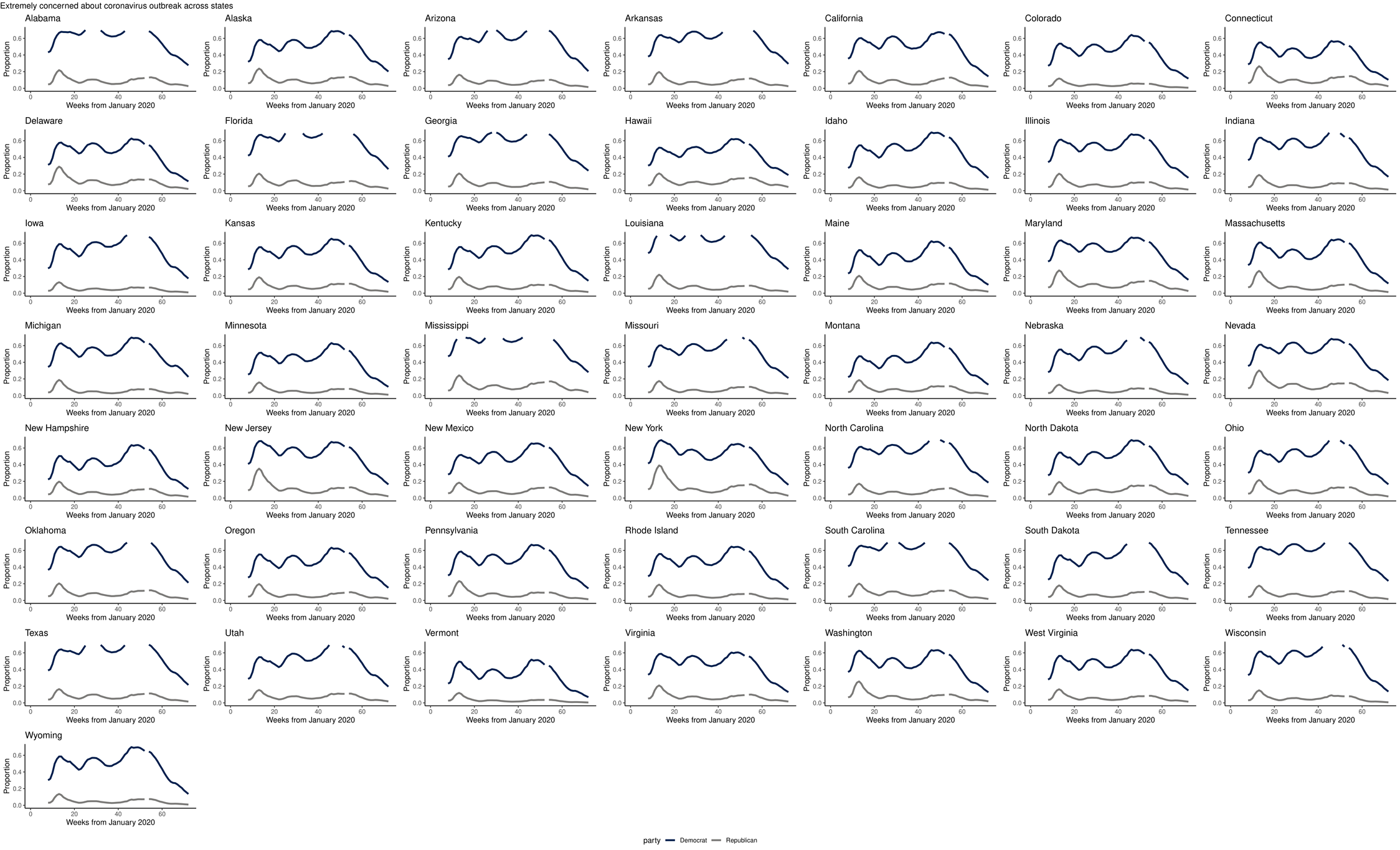

Supplement: S5 Fig — x axes are weeks from January 2020, y axes are proportions of those who are extremely concerned about the outbreak. (TIF) [file pone.0274580.s006.tif]

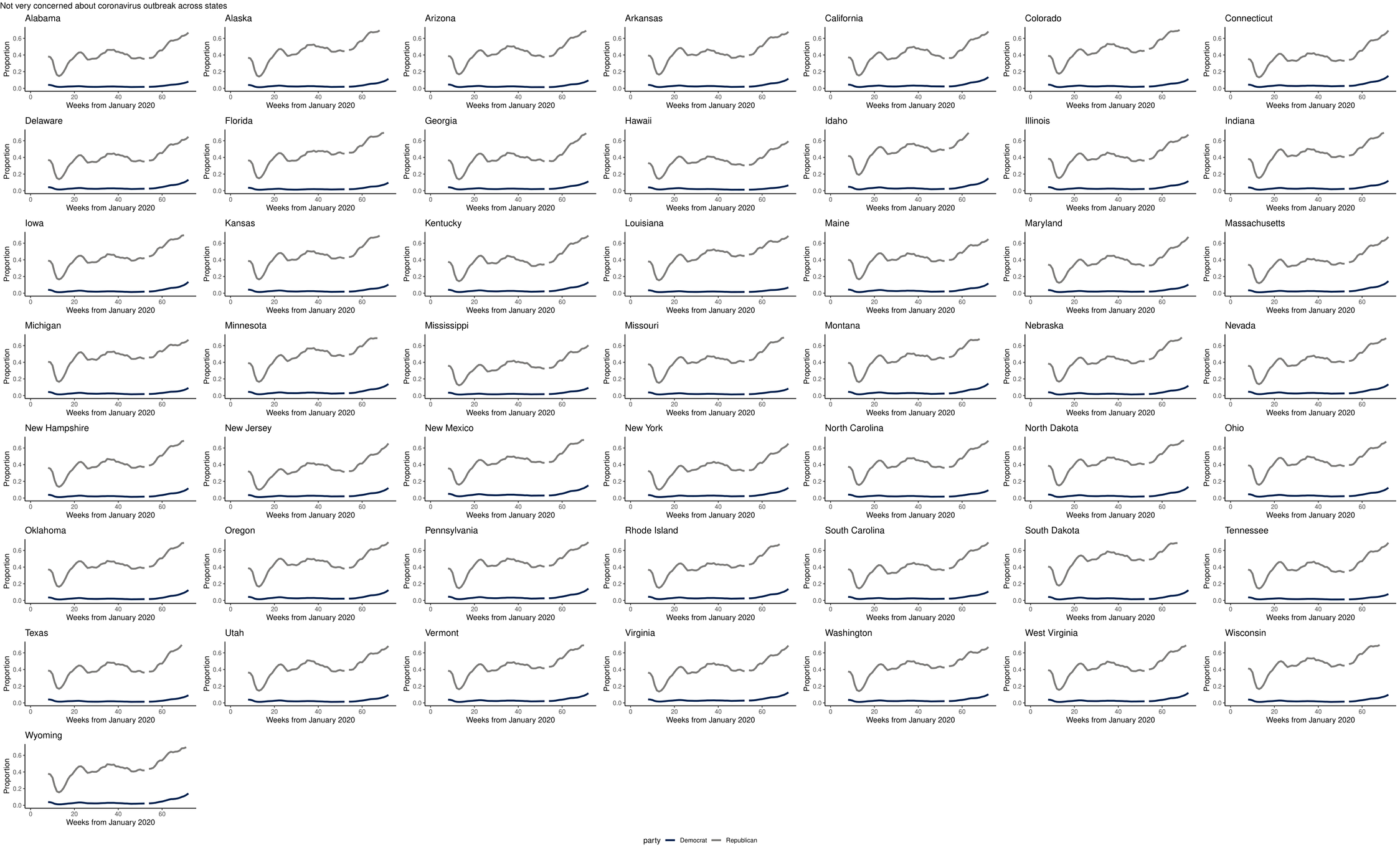

Supplement: S6 Fig — x axes are weeks from January 2020, y axes are proportions of those who are not very concerned about the outbreak. (TIF) [file pone.0274580.s007.tif]

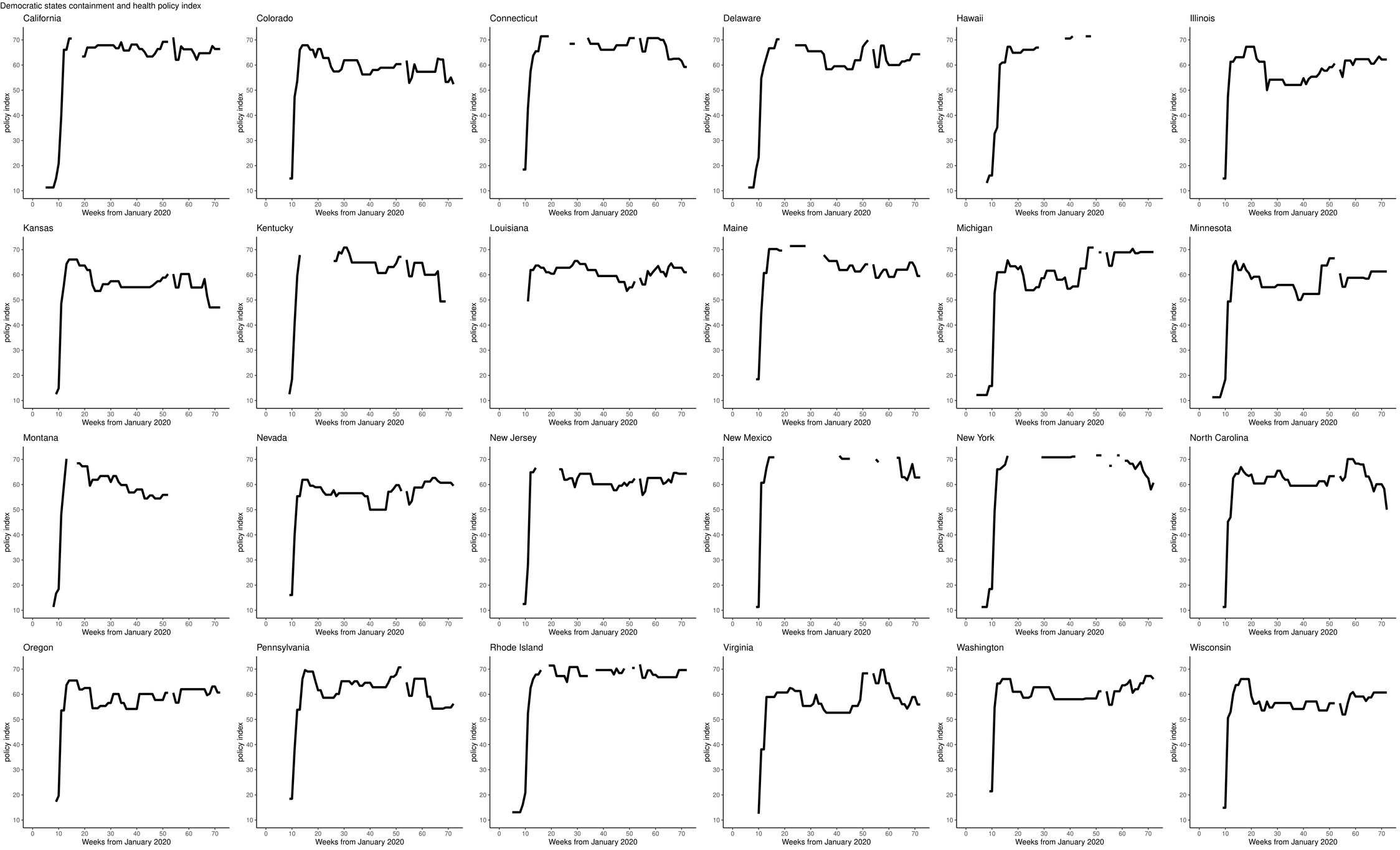

Supplement: S7 Fig — x axes are weeks from January 2020, y axes present policy index. (TIF) [file pone.0274580.s008.tif]

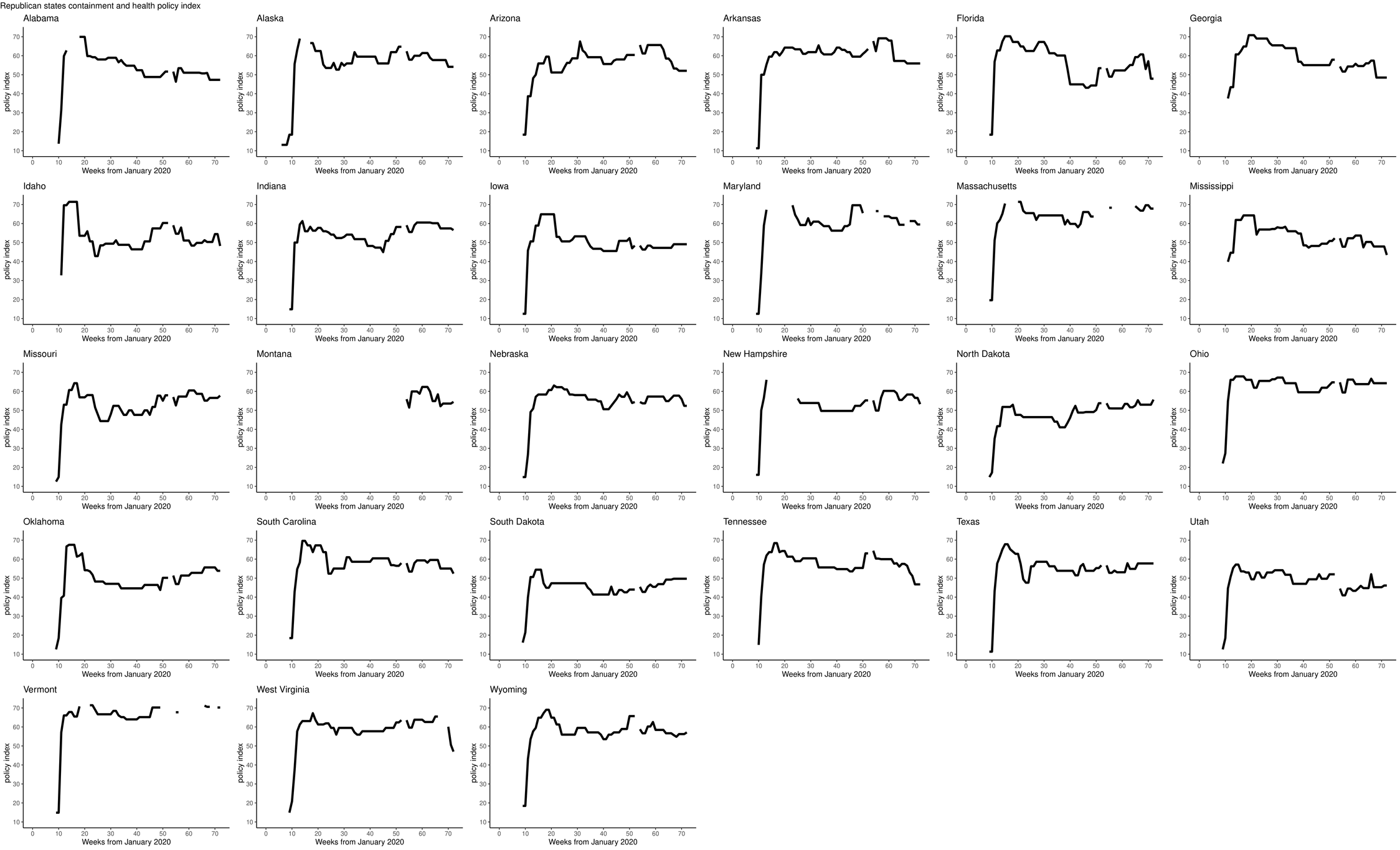

Supplement: S8 Fig — x axes are weeks from January 2020, y axes present policy index. (TIF) [file pone.0274580.s009.tif]

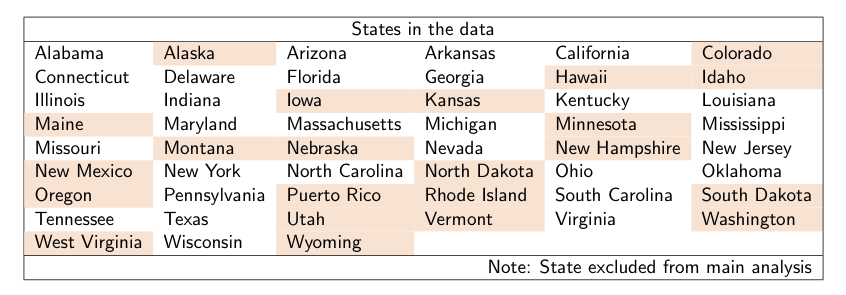

Supplement: S9 Fig — Shaded states include low/suppressed deaths that do not constitute a part of the main analysis as a result. (TIF) [file pone.0274580.s010.tif]
