# Supplementary material for: The polarization of politics and public opinion and their effects on racial inequality in COVID mortality
Source: PLoS One. 2022 Sep 15;17(9):e0274580. doi: 10.1371/journal.pone.0274580 (PMC9477310; doi:10.1371/journal.pone.0274580)
Supplement: S1 File — (PDF) [file pone.0274580.s011.pdf]

Information for study on polarization of politics & public opinion and racial inequality in COVID mortality

Adeline Lo* Héctor Pifarré i Arolas† Jonathan Renshon‡ Siyu Liang§

*Assistant Professor of Political Science, UW-Madison.: aylo@wisc.edu

†Research Director, Centre for Research in Health Economics, Universitat Pompeu Fabra: hector.pifarre@upf.edu

‡Associate Professor of Political Science, UW-Madison. : renshon@wisc.com

§Graduate student of Political Science, UCLA. : sliang46@ucla.edu

Contents

Table of Contents 1

A Summary of data sources 2

B Patterns of COVID-19 mortality 2

C Patterns of public opinions over coronavirus 7

D Patterns of public policies for coronavirus 10

E Summary information on race inequality in mortality variable 13

F Main Model details 13

F.1 Robustness checks on main model 13

G Counterfactuals 14

A Summary of data sources

Several datasets were used in the analysis for this study. The COVID policy data is taken from the Oxford team, "OxCGRT", tracking global government response to the pandemic. In our case, we focused on the U.S. panel coverage of policies data (Hale et al., 2021). The COVID-19 mortality data is from the COVID data tracker, a tracking system published by Center for Disease Control (CDC) that records cases and mortality of the COVID-19 (CDC, 2020). The National Conference of State Legislatures data is used to track information on state governor party and control of state legislature (*Research, Publications, Campaigns and Elections in State Legislatures* 2021). Finally, public opinion for concerns over COVID-19 are collected from the Civiqs American opinion panel research sample, a large nationally representative opt-in online survey panel with daily public opinion tracking on a variety of public opinion questions (Civiqs, 2021); we examined public opinions over the coronavirus pandemic during the period spanning January 2020 to August 2021.

B Patterns of COVID-19 mortality

1. Age adjusted mortality by race in Figure B.1 for the U.S. nationally and by state in Figure B.2.

2. Figure B.3 presents per capita x 100,000 COVID-19 mortality rates for states.

3. Figure D.8 presents the ratio between black and white American COVID-19 mortality per capita.

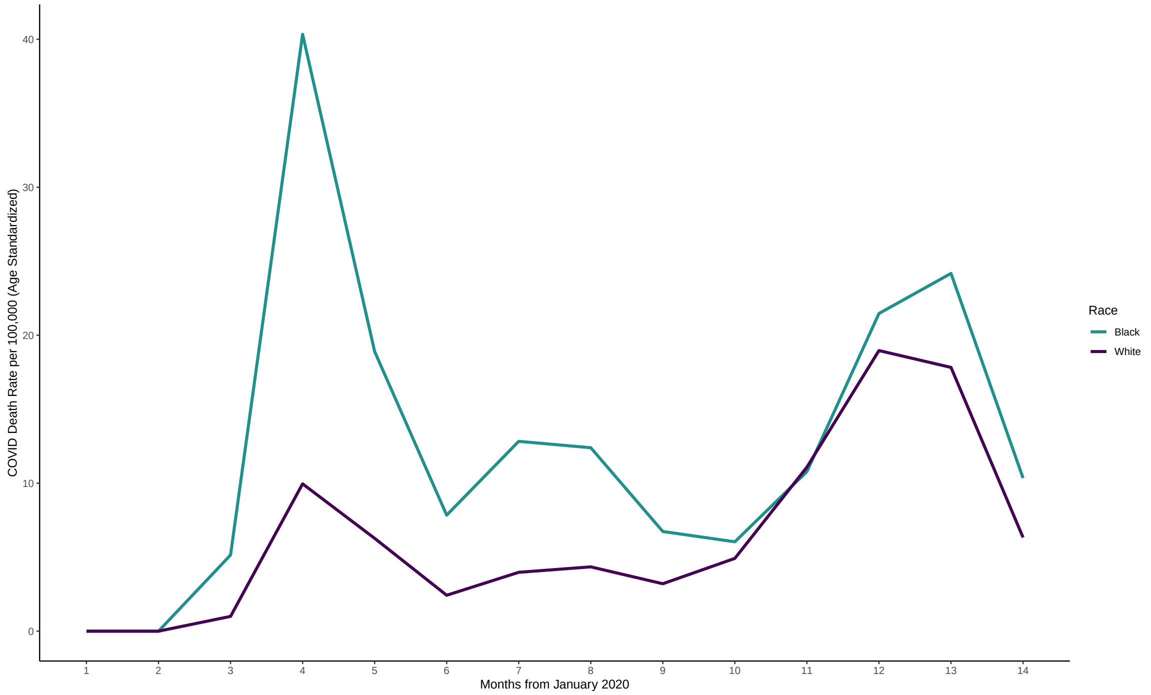

Figure B.1: COVID mortality (aged standardized) for black and white Americans from January 2020 to February 2021. Month since January 2020 in the x-axis; y axis is age standardized COVID death rate.

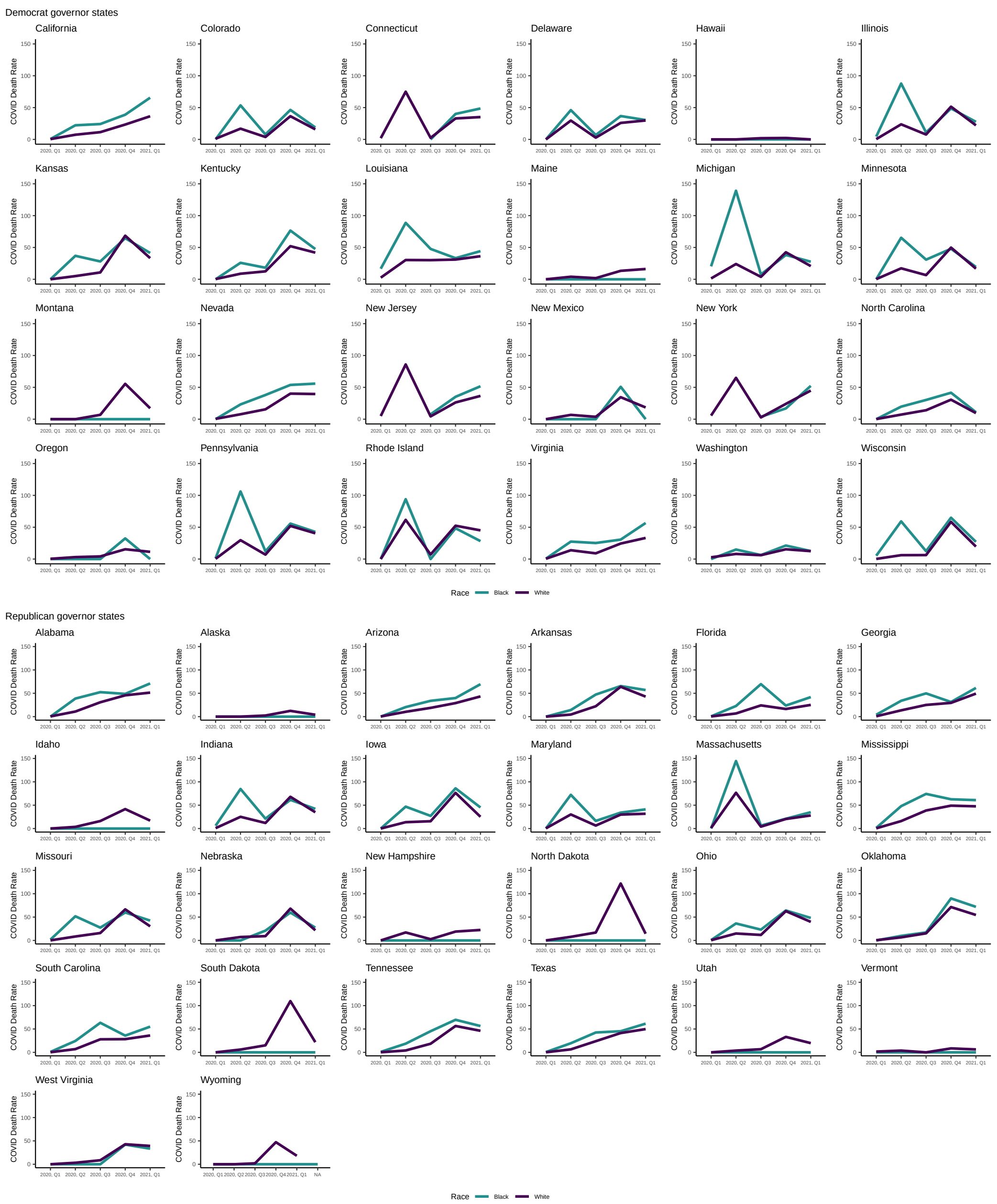

Figure B.2: Age-adjusted COVID mortality, by Democrat and Republican governor states from 2020 Quarter 1 to 2021 Quarter 1. x axes represents year quarter, starting from 2020 Q1 to 2021 Q1; y axes is age standardized COVID death rate.

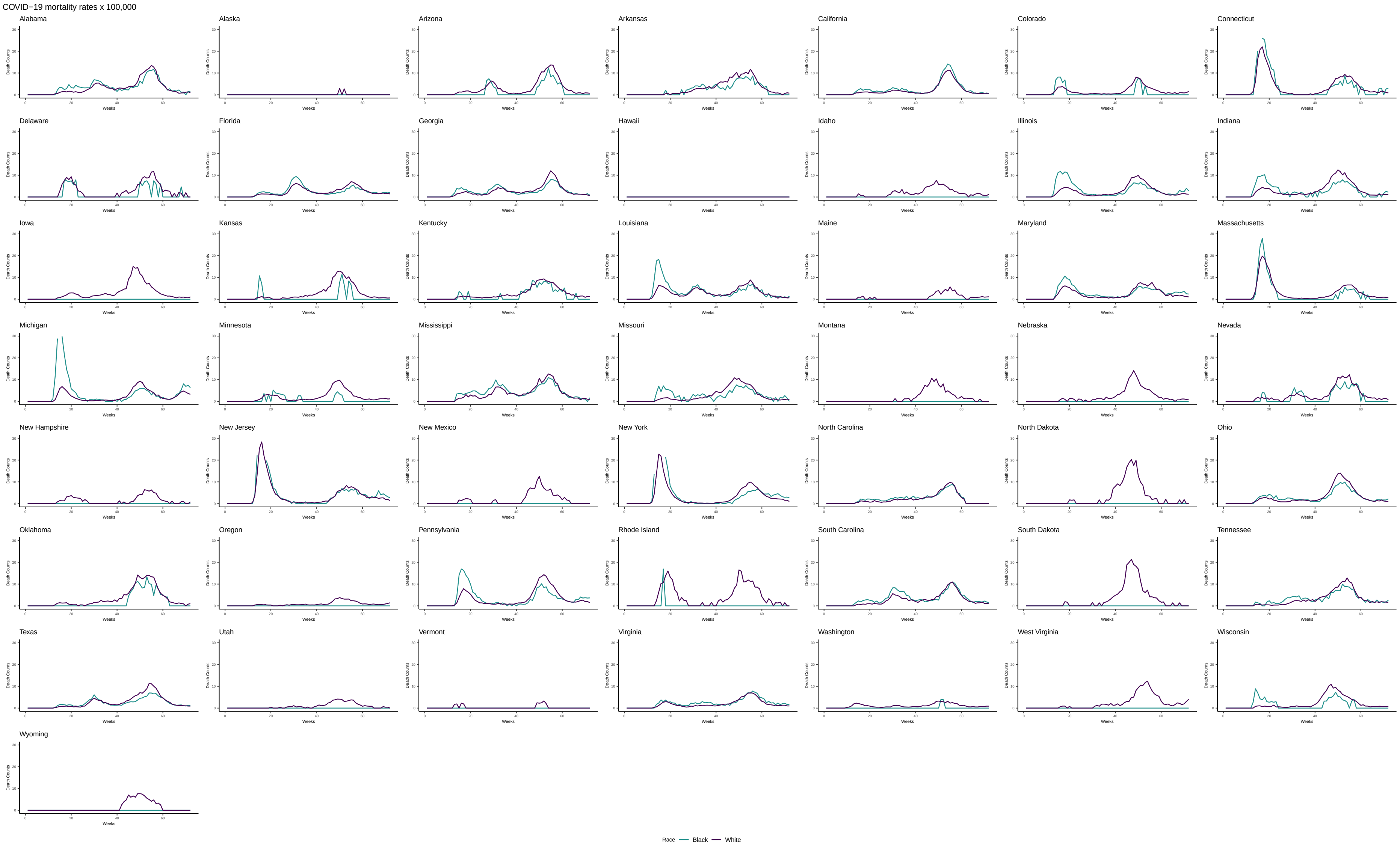

Figure B.3: Per capita times 100,000 COVID-19 mortality rates for states. x axes refers to Weeks from January 2020 , and y axes refers to Covid-19 death counts

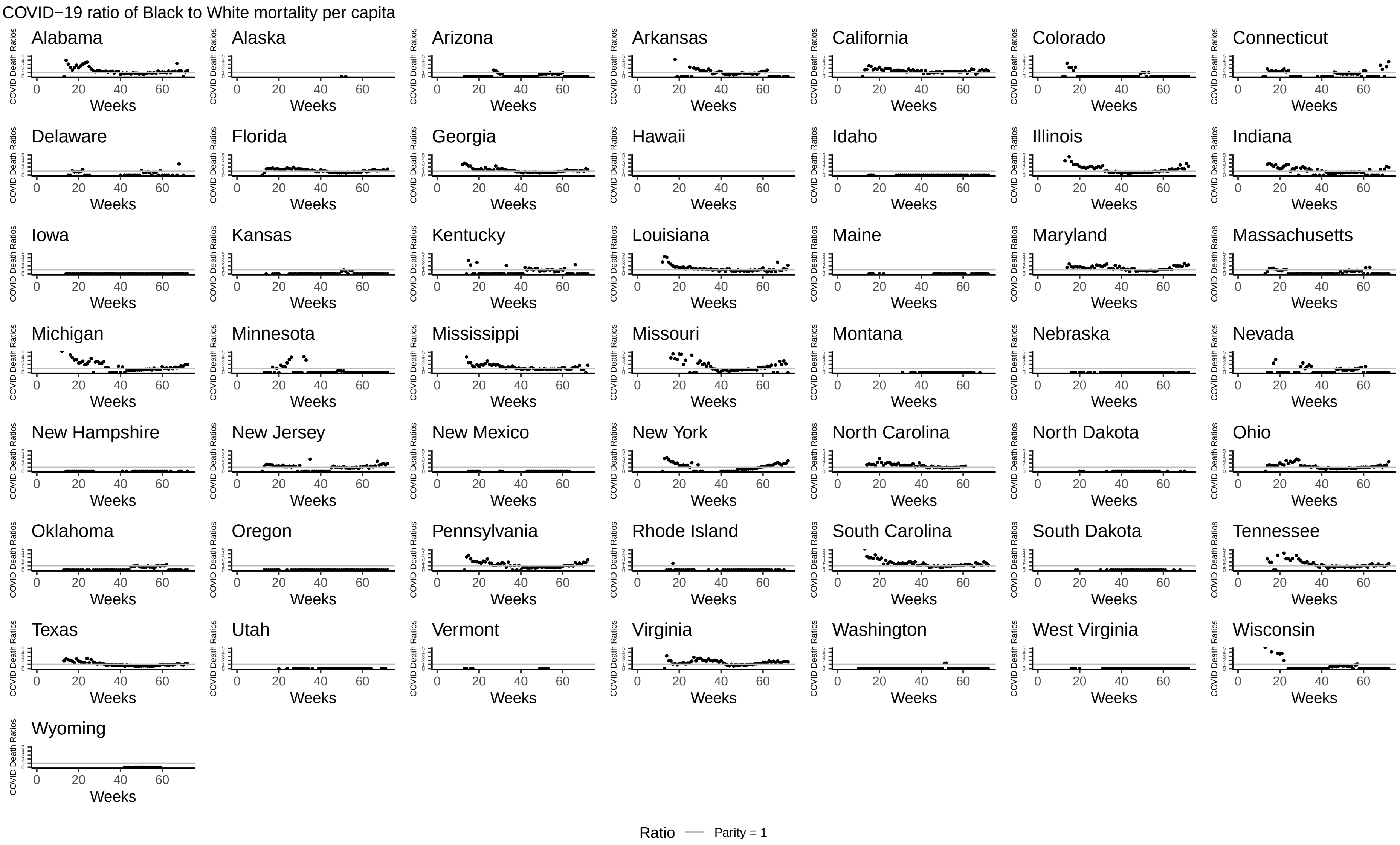

Figure B.4: Ratio between black and white American COVID-19 mortality per capita. x axes are weeks from January 2020, y axes are COVID death ratios.

C Patterns of public opinions over coronavirus

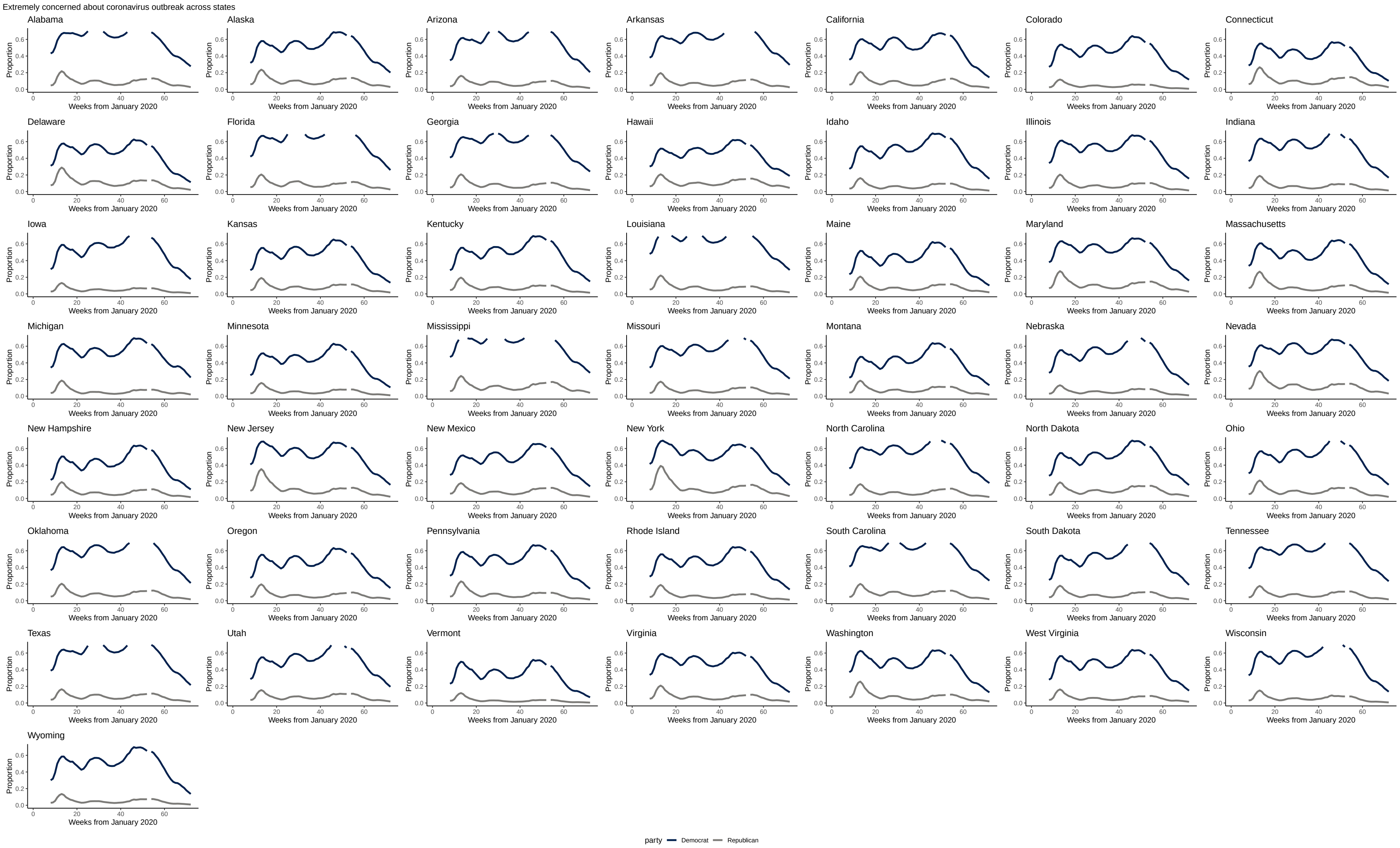

Figure C.5: American who are extremely concerned about the Covid outbreak in the state level
x axes are weeks from January 2020, y axes are proportions of those who are extremely concerned about the outbreak.

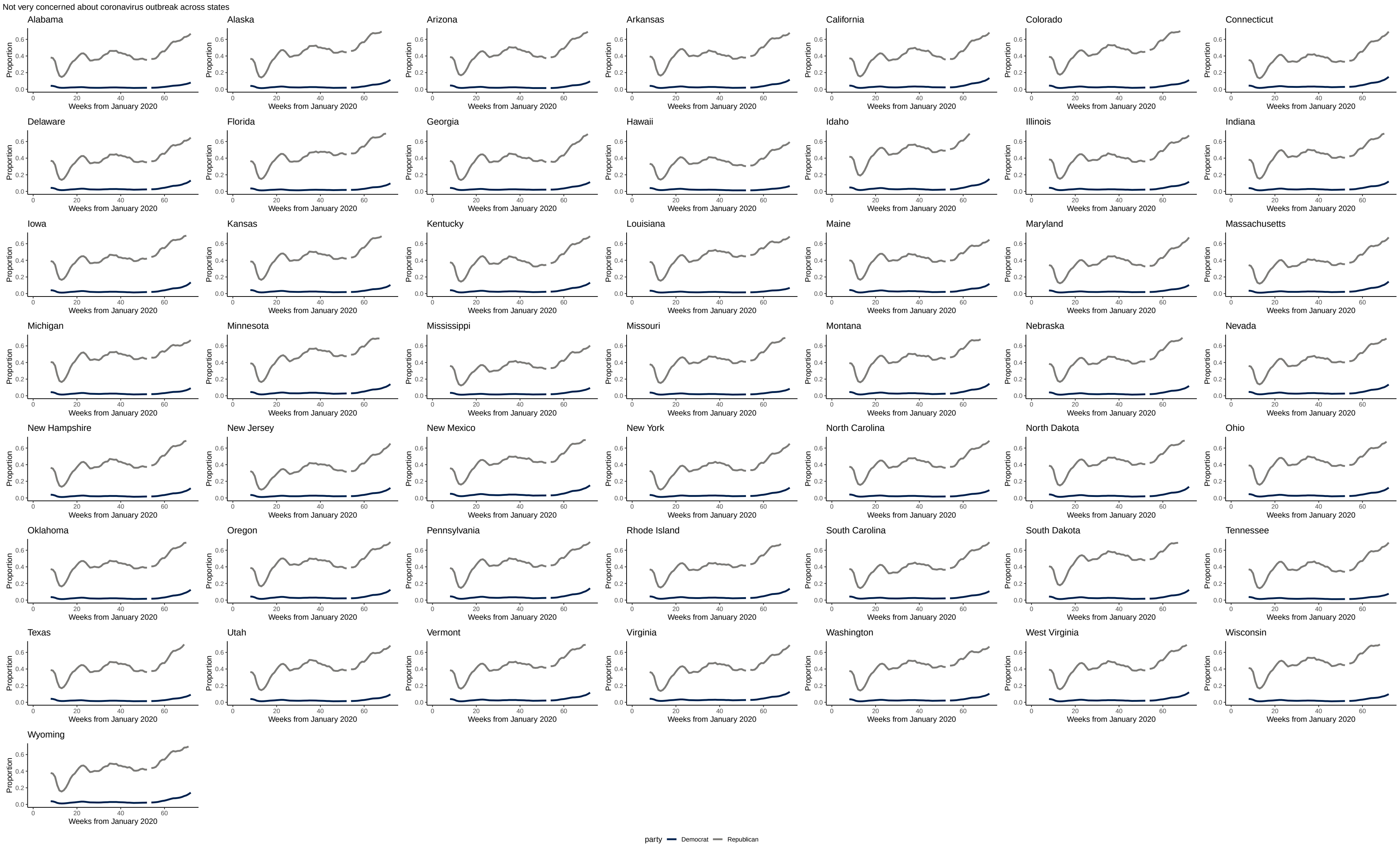

Figure C.6: American who are not very concerned about the Covid outbreak in the state level
x axes are weeks from January 2020, y axes are proportions of those who are not very concerned about the outbreak.

D Patterns of public policies for coronavirus

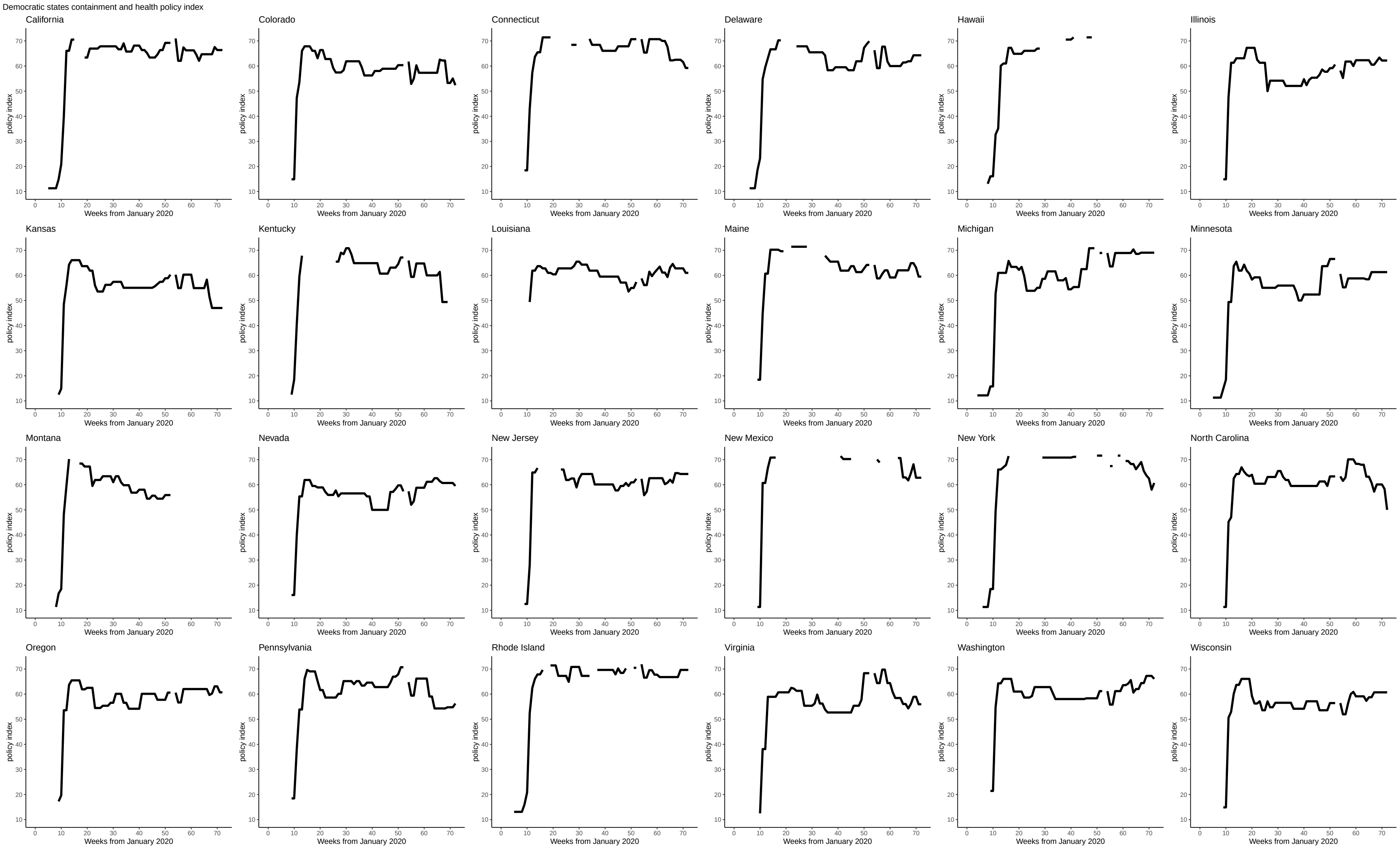

Figure D.7: Containment and health policy adoption in Democratic states
x axes are weeks from January 2020, y axes present policy index.

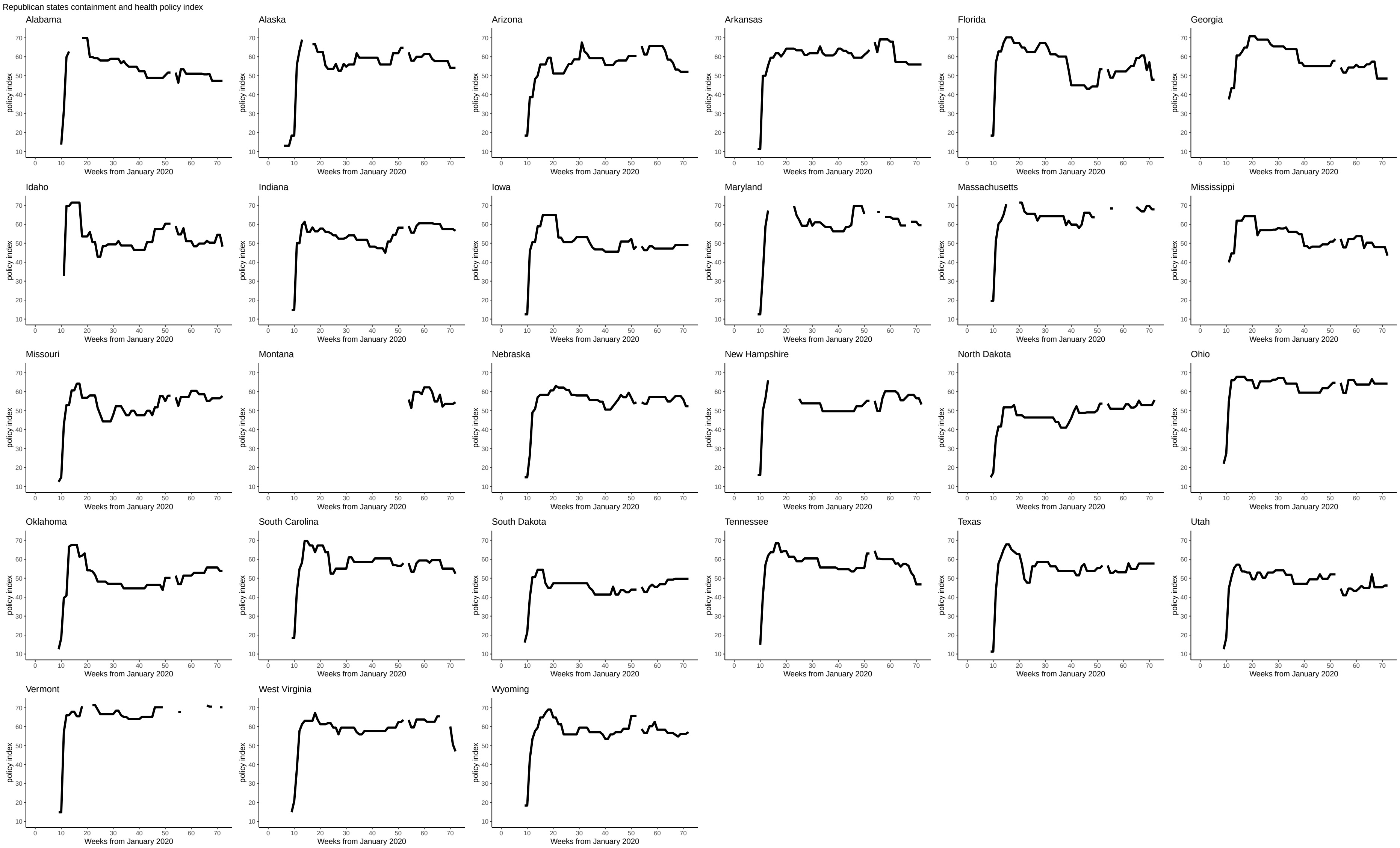

Figure D.8: Containment and health policy adoption in Republican states
x axes are weeks from January 2020, y axes present policy index.

States in the data					
Alabama	Alaska	Arizona	Arkansas	California	Colorado
Connecticut	Delaware	Florida	Georgia	Hawaii	Idaho
Illinois	Indiana	Iowa	Kansas	Kentucky	Louisiana
Maine	Maryland	Massachusetts	Michigan	Minnesota	Mississippi
Missouri	Montana	Nebraska	Nevada	New Hampshire	New Jersey
New Mexico	New York	North Carolina	North Dakota	Ohio	Oklahoma
Oregon	Pennsylvania	Puerto Rico	Rhode Island	South Carolina	South Dakota
Tennessee	Texas	Utah	Vermont	Virginia	Washington
West Virginia	Wisconsin	Wyoming			
					Note: State excluded from main analysis

Figure E.9: All states represented in the full data. Shaded states include low/suppressed deaths that do not constitute a part of the main analysis as a result.

E Summary information on race inequality in mortality variable

To handle CDC-suppressed COVID death counts, we restrict our analysis to states reporting black American deaths in more than 20% of the total 72 weeks in the panel and exclude states with low underlying proportions of black Americans. Among the remaining X states, a few state-weeks still report NAs due to threshold suppression. We verified that these weeks do not constitute a significant portion of the deaths due to COVID and note that removing such weeks still results in a mean coverage of 91% of total COVID-19 deaths in these exact weeks. We focus on black to white inequalities in this study; black Americans are among key non-white minority groups that have been disproportionately affected by COVID and a group consistently covered in COVID data efforts. (Andrasfay and Goldman, 2021). As such, the outcome variable is a ratio of black-to-white American COVID deaths for each state-week, while total COVID-19 mortality is measured as simply total COVID-deaths in a given state-week.

F Main Model details

We conduct Breusch-Pagan, F, and Breusch-Godfrey tests for serial correlation, cross-sectional correlation, and heteroskedasticity in errors and find evidence of all three; the main model accordingly estimates state and time fixed effects, with Driscoll & Kraay robust (to cross-sectional and serial correlation) standard errors.

F.1 Robustness checks on main model

Table F.1 presents the main model (Model 1) and a model that further controls for the inequality between black and white American concern over a local COVID outbreak (Model 2). We also conduct robustness checks where the the containment and health policies index measure is constructed from a rolling average of three or four weeks; our results remain

substantively the same.

	Dependent variable:	
	Ratio of Black to White COVID mortality	
	(Model 1)	(Model 2)
Total COVID-19 mortality	-0.022^{*}	-0.015
	(0.012)	(0.015)
	$p = 0.073$	$p = 0.320$
Ratio of Black-to-White with No Concern for COVID		-4.658
		(7.513)
		$p = 0.536$
Ratio of Black-to-White with High Concern for COVID		0.587^{**}
		(0.276)
		$p = 0.034$
Ratio of Democrat-to-Republicans with No Concern for COVID	13.968^{***}	20.572^{***}
	(4.464)	(7.607)
	$p = 0.002$	$p = 0.007$
Ratio of Democrat-to-Republicans with High Concern for COVID	-0.091	-0.153^{*}
	(0.083)	(0.092)
	$p = 0.274$	$p = 0.095$
Containment & health policy index	-0.012^{**}	-0.013^{**}
	(0.005)	(0.005)
	$p = 0.013$	$p = 0.015$
State FE	Yes	Yes
Week FE	Yes	Yes
Observations	1,225	1,225
F Statistic	12.003^{***} (df = 4; 1131)	11.517^{***} (df = 6; 1129)
Note:		*p<0.1; **p<0.05; ***p<0.01

Table F.1: Ratio of Black to White COVID mortality

G Counterfactuals

We detail our counterfactual thought experiments here. For each week-state, we shift the independent variable according to the experiment in question (e.g. if Democrat governors enacted containment and health policies in similar ways to their Republican colleagues, and if the number of Republican Americans (compared to Democrats) who were "unconcerned" about coronavirus increased by 10%) and predict a counterfactual outcome ($Y_{\text{counterfactual}}$) and compare this against a baseline prediction outcome (Y_{baseline}, or the model prediction of Y with observed independent variables), with the formula below:

Calculations for counterfactuals are aggregated to the average across states of the effect, and compared to the mean predicted level observed using Equation 1.

$$\frac{Y_{\text{counterfactual}} - Y_{\text{baseline}}}{Y_{\text{baseline}}} \times 100 \qquad (1)$$

References

Andrasfay, Theresa and Noreen Goldman (Feb. 2021). “Reductions in 2020 US life expectancy due to COVID-19 and the disproportionate impact on the Black and Latino populations”. *Proceedings of the National Academy of Sciences* 118.5.

CDC (Mar. 2020). *COVID Data Tracker*.

Civiqs (Aug. 2021). *Coronavirus: Outbreak concern*. publisher: Kos Media, LLC, dba Civiqs.

Hale, Thomas, Noam Angrist, Rafael Goldszmidt, Beatriz Kira, Anna Petherick, Toby Phillips, Samuel Webster, Emily Cameron-Blake, Laura Hallas, Saptarshi Majumdar, and Helen Tatlow (Apr. 2021). “A global panel database of pandemic policies (Oxford COVID-19 Government Response Tracker)”. *Nature Human Behaviour* 5.4, pp. 529–538.

Research, Publications, Campaigns and Elections in State Legislatures (2021).
